# Assessing Child Abuse Hotline Inquiries in the Wake of COVID-19: Answering the Call

**DOI:** 10.1001/jamapediatrics.2021.0525

**Published:** 2021-05-03

**Authors:** Robin Ortiz, Rachel Kishton, Laura Sinko, Michelle Fingerman, Diane Moreland, Joanne Wood, Atheendar Venkataramani

**Affiliations:** 1 National Clinician Scholars Program, University of Pennsylvania, Philadelphia; 2 Children’s Hospital of Philadelphia, Philadelphia, Pennsylvania; 3 Childhelp National Child Abuse Hotline, Scottsdale, Arizona; 4 Southwest Interdisciplinary Research Center, Arizona State University, Tempe; 5 Perelman School of Medicine, University of Pennsylvania, Philadelphia

## Abstract

This cross-sectional study assesses inquiries to a child distress hotline during the COVID-19 pandemic compared with inquiries during the same period the previous year.

Experts are concerned about increasing child distress and maltreatment alongside decreasing exposure to mandated child abuse reporters, such as teachers, during the COVID-19 pandemic.^1^ Hotlines may serve as alternate means to identify family violence and support at-risk children. This study assessed the volume of calls and texts to a national child abuse hotline during the pandemic compared with the prior year.

## Methods

This cross-sectional study was conducted using restricted-access data from Childhelp, the only national hotline with a primary focus on child abuse and neglect. Childhelp has offered 24-hour multilingual counseling across all US states via phone call inquiries from youth and concerned adults since 1982 and via text message since 2019.^2,3^ Users anonymously provide optional demographic information, including their relationship to the youth (eg, themselves, parent, neighbor, or teacher). Users are then connected to a crisis counselor. Study data included the number of inquiries, modality (call or text), and demographic characteristics (inquirer’s age category, sex, and identifier type). The University of Pennsylvania Institutional Review Board deemed this study nonhuman subjects research.

Given the initiation of school closures on March 5, 2020, we examined differences in demographic information by modality between March 1, 2019, and May 27, 2019, and March 1, 2020, and May 26, 2020, using χ^2^ and Fischer exact tests. We then assessed inquiries for each full year beginning in January by modality. Analyses were performed using Stata/IC version 15.1 (StataCorp), with 2-tailed significance set at *P* < .05.

## Results

From March to May in 2019 and 2020 combined, Childhelp received 35 480 call and text inquiries, mostly from female individuals (74.63%) and adults 18 years and older (92.97%) (Table). Nearly 96% of callers were adults (18 years and older), while most texters were younger than 18 years. There was a 13.75% increase in the total number of inquiries in 2020 compared with 2019. Caller type differed between 2020 and 2019 as well, with a decrease in calls from school reporters (teachers, school personnel, and daycare personnel) and a smaller decrease from non–school-based mandated reporters (Child Protective Services [CPS] workers, counselors, foster care providers, health care workers, and authorities). There was an increase in calls from neighbors or landlords, relatives, and friends, and other caller types remained relatively stable (within 1%).

**Table.** Characteristics of Hotline Inquiries and Users From March to May in 2019 and 2020

Characteristic	No. (%)		*P* value
	2019 (n = 16 599)	2020 (n = 18 881)	
Call	16 299	17 618	NA
Text	300	1263	NA
**Callers**			
Adult (≥18 y)	13 277 (95.69)	14 211 (95.54)	.54
Youth (<18 y)	598 (4.30)	663 (4.46)	
Male	3371 (24.45)	3867 (26.09)	.006
Female	10 401 (75.47)	10 942 (73.84)	
Caller type			<.001
Total, No.	13 901	14 912	
Individual experiencing abuse	375 (2.70)	379 (2.54)	
Parent or guardian	2982 (21.50)	3297 (22.11)	
School reporter	498 (3.56)	316 (2.12)	
Non–school-based mandated reporter	960 (6.91)	936 (6.28)	
Neighbor or landlord	721 (5.19)	1293 (8.67)	
Relative	1599 (11.50)	2126 (14.26)	
Friend	706 (5.08)	949 (6.36)	
Bystander	151 (1.09)	294 (1.97)	
Other individual experiencing abuse	388 (2.79)	545 (3.65)	
Other individual reporting abuse	71 (0.51)	54 (0.36)	
Other or unknown	5450 (39.21)	4723 (31.72)	
**Texters**			
Adult (≥18 y)	53 (19.92)	452 (41.32)	<.001
Youth (<18 y)	213 (80.10)	642 (58.68)	
Male	75 (29.18)	240 (21.78)	.03
Female	179 (69.65)	837 (75.95)	
Texter type			<.001
Total, No.	286	1153	
Individual experiencing abuse	149 (52.10)	346 (30.09)	
Parent or guardian	11 (3.85)	85 (7.11)	
School reporter	1 (0.35)	4 (0.35)	
Non–school-based mandated reporter	1 (0.35)	6 (0.52)	
Neighbor or landlord	2 (0.70)	61 (5.29)	
Relative	8 (2.80)	64 (5.55)	
Friend	42 (14.70)	180 (15.61)	
Bystander	5 (1.75)	37 (3.21)	
Other individual experiencing abuse	8 (2.80)	198 (17.17)	
Other individual reporting abuse	0	0	
Other or unknown	59 (20.63)	172 (14.92)	

Abbreviation: NA, not applicable.

Calls increased after the declaration of a health emergency by the US Secretary of Health and Human Services on January 31, 2020, but then decreased after the initiation of school closures on March 5, 2020 (Figure). Additional analyses (not shown) revealed that a sustained decrease in school reporter calls was contrasted by a later increase in parent calls. Text inquiries increased after initial school closures. In May 2020, both calls and texts surged higher than 2019 levels.

**Figure.**
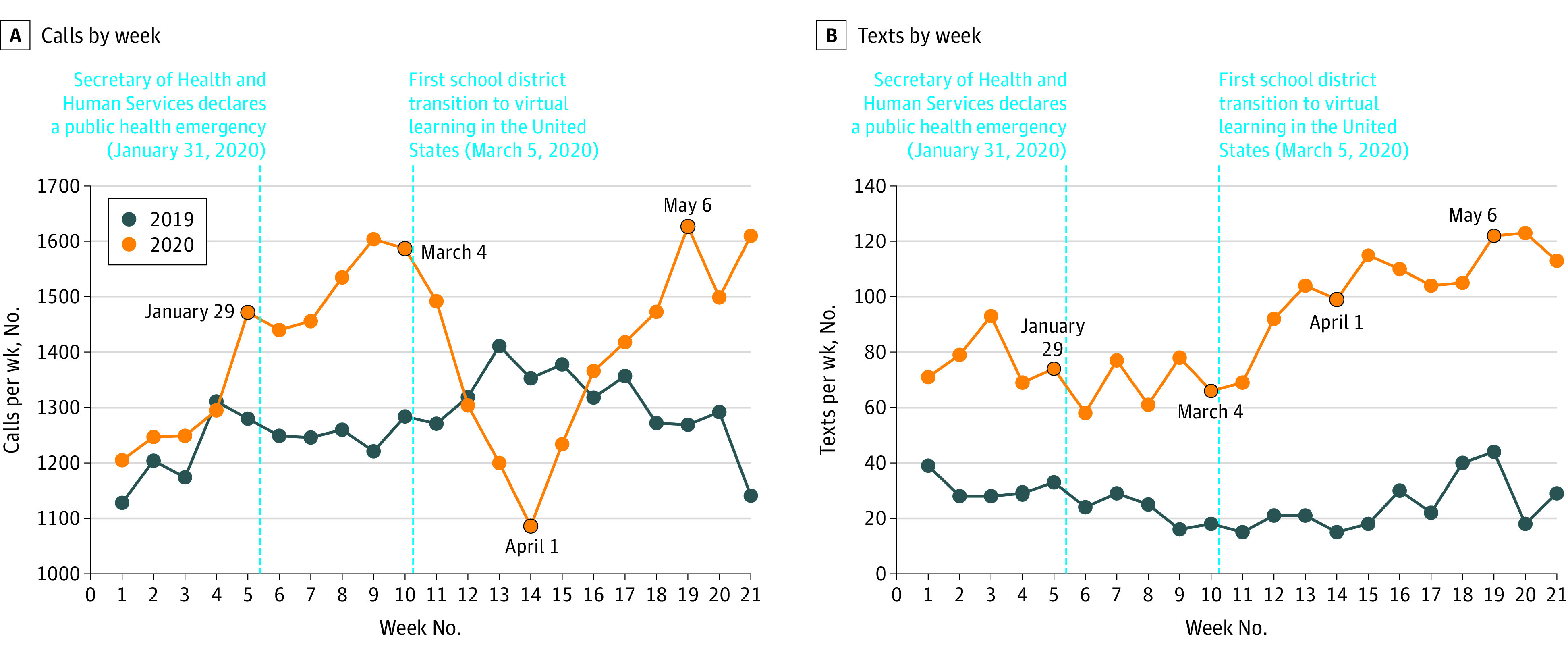
Child Abuse Hotline Calls and Text Messages by Week in 2019 and 2020 Phone call and text inquiries are plotted as number of inquiries per week between January 1, 2019, and May 27, 2019, and January 1, 2020, and May 26, 2020. The vertical dashed line indicates the declaration of a health emergency by the Secretary of Health and Human Services, which occurred on January 31, 2020. The vertical dotted line indicates the initiation of school closures, which began on March 5, 2020. Calls initially increased, then decreased after January 31, 2020, and then increased again in May 2020 above 2019 levels. Text inquiries in 2020, while not directly comparable with 2019 data given their novelty as an added modality that year, more steadily increased after March 5, 2020, compared with phone calls.

## Discussion

In this national study, overall inquiries by phone call and text message to a child abuse hotline increased following school closures and quarantine orders associated with the COIVD-19 pandemic compared with overall inquiries in 2019. This may reflect a higher rate of child-related distress and maltreatment. After a dramatic decrease in calls during the immediate postclosure period, call volume rebounded by May 2020, and use of texts steadily increased. Decreased exposure to school-based mandated reporters may have contributed to the initial call decrease. Text messaging, a child- and teenager-friendly modality, expanded during the postclosure period, pointing to potential self-advocacy.

Limitations of this study include the inability to assess the nature or duration of user concerns, detailed demographic information, and generalizability to all children or CPS report volumes amid the pandemic. Nevertheless, our findings suggest that text-based access to hotlines or agencies may be an effective strategy while exposure to mandated reporters, particularly school personnel, remains limited. Our findings also suggest that hotline data, including both call and text inquiries, may provide a novel source of information to investigate the effect of COVID-19 on a vulnerable population otherwise challenging to study.
